# Cardiac-Specific Inhibition of Kinase Activity in Calcium/Calmodulin-Dependent Protein Kinase Kinase-β Leads to Accelerated Left Ventricular Remodeling and Heart Failure after Transverse Aortic Constriction in Mice

**DOI:** 10.1371/journal.pone.0108201

**Published:** 2014-09-25

**Authors:** Shin Watanabe, Takahiro Horie, Kazuya Nagao, Yasuhide Kuwabara, Osamu Baba, Hitoo Nishi, Naoya Sowa, Michiko Narazaki, Tetsuya Matsuda, Genzou Takemura, Hiromichi Wada, Koji Hasegawa, Takeshi Kimura, Koh Ono

**Affiliations:** 1 Department of Cardiovascular Medicine, Graduate School of Medicine, Kyoto University, Kyoto, Japan; 2 Department of Systems Science, Graduate School of Informatics, Kyoto University, Kyoto, Japan; 3 Department of Internal Medicine, Asahi University, Gifu, Japan; 4 Division of Translational Research, Kyoto Medical Center, National Hospital Organization, Kyoto, Japan; Scuola Superiore Sant’Anna, Italy

## Abstract

**Background:**

The mechanism of cardiac energy production against sustained pressure overload remains to be elucidated.

**Methods and Results:**

We generated cardiac-specific kinase-dead (kd) calcium/calmodulin-dependent protein kinase kinase-β (CaMKKβ) transgenic (α-MHC CaMKKβ^kd^ TG) mice using α-myosin heavy chain (α-MHC) promoter. Although CaMKKβ activity was significantly reduced, these mice had normal cardiac function and morphology at baseline. Here, we show that transverse aortic binding (TAC) in α-MHC CaMKKβ^kd^ TG mice led to accelerated death and left ventricular (LV) dilatation and dysfunction, which was accompanied by significant clinical signs of heart failure. CaMKKβ downstream signaling molecules, including adenosine monophosphate-activated protein kinase (AMPK), were also suppressed in α-MHC CaMKKβ^kd^ TG mice compared with wild-type (WT) mice. The expression levels of peroxisome proliferator-activated receptor-γ coactivator (PGC)-1α, which is a downstream target of both of CaMKKβ and calcium/calmodulin kinases, were also significantly reduced in α-MHC CaMKKβ^kd^ TG mice compared with WT mice after TAC. In accordance with these findings, mitochondrial morphogenesis was damaged and creatine phosphate/β-ATP ratios assessed by magnetic resonance spectroscopy were suppressed in α-MHC CaMKKβ^kd^ TG mice compared with WT mice after TAC.

**Conclusions:**

These data indicate that CaMKKβ exerts protective effects on cardiac adaptive energy pooling against pressure-overload possibly through phosphorylation of AMPK and by upregulation of PGC-1α. Thus, CaMKKβ may be a therapeutic target for the treatment of heart failure.

## Introduction

Previous studies determined that a calcium (Ca^2+^)-mediated signaling cascade resulting from mechanical overload or Gq-mediated signaling initiates changes that lead to cardiac hypertrophy through the activation of calcineurin and consequent targeting of nuclear factor of activated T-cells (NFAT) transcription factors. Ca^2+^/calmodulin kinase (CaMK) II activation and subsequent NFAT3 signaling act in concert to promote pathologic hypertrophic signaling and cardiac growth. However, there is a lack of knowledge whether there is a signaling mechanism to compensate for cardiac energy production against sustained pressure load.

Numerous hormones, growth factors, and physiological processes cause a rise in cytosolic Ca^2+^ concentration, which is translated into cellular responses by interacting with a large number of Ca^2+^-binding proteins [1]. The Ca^2+^-binding protein that is most pervasive in mediating these responses is calmodulin (CaM), which acts as a primary receptor for Ca^2+^ in all eukaryotic cells [2]. Upon Ca^2+^ binding, CaM increases its affinity for a large number of CaM-binding proteins, including three multifunctional CaM kinases (CaMKs; CaMKI, CaMKII, and CaMKIV). CaMK kinases (CaMKKs) initiate the signaling cascade by phosphorylation and activation of two CaMKs, CaMKI and CaMKIV, whereas CaMKII can be activated by Ca^2+^/CaM without the activation of CaMKK [3]. Two CaMKK genes (CaMKKα and CaMKKβ) have been identified in mammals, both of which are strongly expressed in the brain [4], [5]. For full activation, CaMKI and CaMKIV require phosphorylation on an activation loop Thr by CaMKKα or CaMKKβ. In addition to its role in these enzymatic cascades, CaMKKβ is also a physiologically relevant upstream activator of adenosine monophosphate (AMP)-activated protein kinase (AMPK); this CaMKKβ-AMPK complex is known to regulate the energy balance by acting in the hypothalamus [6]. We demonstrated previously that CaMKKβ is important for GLUT4 translocation through AMPK activation in cardiomyocytes [7]. Moreover, CaMKKβ was shown to be important for mitochondrial biogenesis and exercise tolerance through its downstream target of peroxisome proliferator-activated receptor-γ coactivator (PGC)-1α by the use of muscle-specific adiponectin-deficient mice [8]. Therefore, it is possible that CaMKKβ in the heart exerts its role to compensate cardiac energy production against Ca^2+^ overload induced by sustained pressure load.

In this study, we focused on CaMKKβ in the control of cardiac function after transverse aortic constriction (TAC). We generated cardiac-specific kinase-dead (kd) CaMKKβ (CaMKKβ^kd^) transgenic (TG) mice, using an α-myosin heavy chain (α-MHC) promoter to define the structural and functional responses of the left ventricle to pressure-overload stress in the absence of an intact CaMKKβ cascade.

## Materials and Methods

### 1. Mice

Male C57BL/6 mice were purchased from Japan SLC inc. (Shizuoka, Japan) and maintained in a specific pathogen-free facility. The α-MHC promoter drives transgene expression exclusively in cardiac myocytes and has been used extensively in transgenic studies. A kd form of CaMKKβ harboring a mutation of aspartic acid residue 329 to alanine had no effect on its downstream kinases [9] was utilized in our previous experiments [7]. To generate α-MHC CaMKKβ^kd^ TG mice, we placed an α-MHC promoter and FLAG tag at the 5′ end of a kd form of CaMKKβ. Transgenic DNA was microinjected into zygotes using a standard methodology, as described previously [10]. Transgenic mice and non-transgenic littermates as controls were maintained on a 12-h light/dark cycle, fed a normal laboratory diet *ad libitum*, sacrificed by decapitation under ether anesthesia at the indicated age and analyzed. This investigation conformed to the Guide for the Care and Use of Laboratory Animals published by the US National Institutes of Health (NIH Publication No. 85-23, revised 1996). All animal care, experiments, and methods were approved by the Animal Care and Use Committees of Kyoto University Graduate School of Medicine. We determined humane endpoints beforehand for the survival study. The criteria used to determine when the animals should be humanely sacrificed is rapid or progressive weight loss of more than 20% of the body weight, dehydration determined by an increase in skin tenting, sunken eyes, respiratory symptoms such as labored breathing, nasal discharge, coughing, or cyanosis. The animals were carefully monitored three times a day.

### 2. Surgery

Constriction of the transverse thoracic aorta was performed on 3-month-old mice as described previously [11]. In brief, mice were anesthetized, intubated, and placed on a respirator. Midline sternotomy was performed, the aorta was visualized, and a 6.0 prolene suture was placed around the aorta distal to the brachiocephalic artery. The suture was tightened around a blunt 26-gauge needle placed adjacent to the aorta. The needle was then removed, and the chest and overlying skin were closed. Sham-operated mice underwent a similar operation in which the aortic arch was isolated and a band was tightened around the aorta but not ligated and was removed subsequently.

### 3. Transthoracic echocardiography

Echocardiography was performed as described previously [12]. Briefly, mice were anesthetized using an intraperitoneal injection of 2-2-2 tribromoethanol (240 mg/kg, Wako Pure Chemical Industries, Osaka, Japan), and transthoracic echocardiography was performed using a Sonos-5500 echocardiograph (Agilent Technologies, Santa Clara, CA) with a 15-MHz linear transducer. M-mode echocardiograms were obtained at the papillary muscle level. At least two independent M-mode measurements per animal were carried out by an examiner blinded to the genotype of the animals. Fractional shortening was calculated as (LVIDd–LVIDs)/LVIDd*100.

### 4. Histological analysis

After anesthesia, we injected 0.1 mL of 1% CdCl_2_ via inferior vena cava to achieve diastolic arrest. Then we perfused and fixed mice with 4% paraformaldehyde at 30 cm H_2_O before excising the heart, which was further fixed in 4% paraformaldehyde at 4°C overnight. Paraffin sections were stained with Masson trichrome and Sirius red staining. All photos were taken using a BZ-9000 microscope (Keyence, Osaka, Japan).

### 5. Transmission electron microscopy

Left ventricular (LV) cardiac tissue was quickly cut into 1 mm cubes, immersion-fixed with 2.5% glutaraldehyde in 0.1 mol/L phosphate buffer (pH 7.4) overnight at 4°C, and postfixed in 1% buffered osmium tetroxide. The specimens were then dehydrated through a graded ethanol series and embedded in epoxy resin. Ultrathin sections (90 nm), double-stained with uranyl acetate and lead citrate, were examined using an electron microscope (H-800, Hitachi, Tokyo, Japan).

### 6. Mitochondrial DNA analysis

The amount of mitochondrial DNA was quantified using quantitative PCR as described previously [13]. Total DNA was isolated using a QIAmp DNA microkit (Qiagen, Venlo, Netherlands). The levels of cytochrome b (*Cytb*), NADH dehydrogenase subunit 1 (*Nd1*), and cytochrome c oxidase 1 (*Co1*) genes in the mtDNA genome were quantified. Results were normalized to the nuclear housekeeping gene H19. The delta-delta Ct method was used for quantification. Primer sequences are indicated in Table S1.

### 7. Measurement of superoxide

Superoxide levels were measured using a superoxide-sensitive dye, dihydroethidine (DHE). DHE is cell permeable and, in the presence of superoxide, is converted to fluorescent ethidium bromide (EtBR), which is trapped by intercalating with DNA. 5 µM DHE (Sigma-Aldrich, St. Louis, MO) was applied to heart cross-sections (7 µm) and incubated in a light-protected and humidified chamber at 37°C for 30 min. In situ fluorescence was assessed using fluorescence microscopy.

### 8. Western blotting

Total protein was extracted from frozen hearts, resolved and subjected to sodium dodecylsulfate polyacrylamide gel electrophoresis (SDS-PAGE) followed by standard Western blotting procedures [7]. A total of 20 µg protein was fractionated using NuPAGE 4–12% Bis-Tris (Invitrogen) gels and transferred to a Protran nitrocellulose transfer membrane (Whatman). The antibodies used for Western blotting were as follows: anti-CaMKKβ (H-95) (Santa Cruz Biotechnology, Dallas, TX, USA), 1∶500; anti-total-CaMKI (sc-33165) (Santa Cruz Biotechnology), 1∶1000; anti-phospho-CaMKIV (sc-28443-R) (Santa Cruz Biotechnology), 1∶500; anti-total-CaMKIV (sc-55501) (Santa Cruz Biotechnology), 1∶1000; anti-phospho-AMPKα (Cell Signaling Technology, Beverly, MA, USA), 1∶500; anti-total-AMPKα (Cell Signaling Technology), 1∶1000; and anti-GAPDH (Cell Signaling Technology), 1∶2000. Anti-phospho-CaMKI (Thr177) was a kind gift from Dr. Naohito Nozaki (Kanagawa Dental College, Yokosuka, Kanagawa, Japan) and used at the dilution of 1∶50. Anti-rabbit IgG (GE Healthcare) and anti-mouse IgG (GE Healthcare) were used as secondary antibodies at a dilution of 1∶2000.

### 9. CaMKKβ activity

Heart tissues were rapidly frozen in liquid nitrogen. The frozen tissue was ground and resuspended in 100 µL homogenization buffer (50 mM Tris/HCl, 0.25 nM mannitol, 50 mM NaF, 5 mM sodium pyrophosphate, 150 mM NaCl, 5 µg/mL soybean trypsin inhibitor, 0.1 mM PMSF, 1% (v/v) Triton X-100) per 20 mg of tissue and further homogenized using a hand-held motor driven pestle. The samples were left on ice for 30 min, and then cell debris was pelleted by centrifugation. The supernatants were removed and protein concentrations were determined. CaMKKβ was immunoprecipitated from 500 µg of protein for 2 h at 4°C with 10 µl of anti CaMKKβ antibody (H-95, Santa Cruz Biotechnology). The immunoprecipitates were washed and then resuspended in kinase buffer containing 25 µM ATP and 5 µCi of [γ-^32^P]ATP in the presence of 1 mM calcium and plus 1 µM calmodulin and 0.5 µg of purified recombinant human CAMKI (P01, Abnova Corporation, Taipei, Taiwan) was added and incubated on an orbital shaker at 37°C for 45 min. The reactions were stopped by addition of sample loading dye. The reaction mixtures were separated by SDS-PAGE and exposed to X-ray film after drying the gel. Total immunoprecipitated CaMKKβ protein were analyzed individually by Western blotting.

### 10. Quantitative real-time (q-RT) PCR for mRNA

Total RNA was isolated using TRIzol reagent (Invitrogen, Carlsbad, CA). cDNA was synthesized using SuperScript II reverse transcriptase (Invitrogen) and the PCR reaction was performed using SYBR Green PCR master mix (Applied Biosystems, Foster, CA), normalized with GAPDH. Gene-specific primer sequences are indicated in Table S1.

### 11. Magnetic resonance imaging protocol

Magnetic resonance (MR) imaging experiments were performed as described previously [14]. Briefly, experiments were carried out using a Bruker Biospec MRI/MRS spectrometer (Ettlingen, Germany) equipped with a 7-T/20-cm Oxford magnet and a 12-cm (inner diameter) actively shielded gradient set, as described previously. In vivo MRI/MRS studies were carried out on wild-type (WT) and TG mice before and after TAC. Anesthesia was induced with ∼1% isoflurane, as described previously. The probe set was a 20-mm ^1^H/^31^P MRS coil. Metabolite areas were determined from the integrated peak areas of the creatine phosphate (PCr) and [β-P]ATP resonances from voxels centered on cardiac muscle as identified by high-resolution MR imaging.

### 12. Statistics

Data are presented as means ± standard error (SE). Statistical comparisons were performed using unpaired two-tailed Student’s *t*-tests or one-way analysis of variance with Bonferonni’s post hoc test where appropriate. We sat the level for statistical significance at a p<0.05.

## Results

### 1. CaMKKβ expression is upregulated by TAC

**Figure 1:**
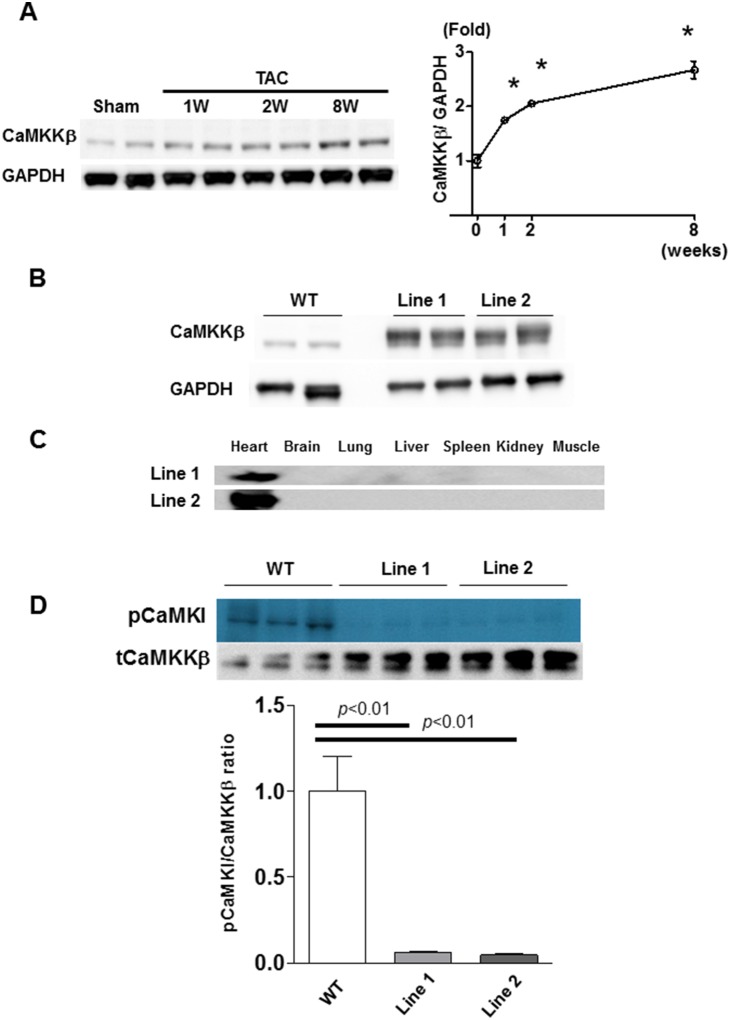
Generation of α-MHC CaMKKβ^kd^ TG mice. A. Immunoblotting and densitometry for calcium/calmodulin-dependent protein kinase kinase-β (CaMKKβ) after transverse aortic binding (TAC) in wild-type (WT) mice. Values are the means ± standard error (S.E.) of four independent experiments (*p<0.05 vs 0 week). B. Immunoblotting for CaMKKβ in WT mice and two lines of α-MHC CaMKKβ^kd^ TG mice. C. Immunoblotting for flag tag in two lines of α-MHC CaMKKβ^kd^ TG mice indicating the heart-specific overexpression of the transgene. D. Activities of CaMKKβ in the hearts of two lines of α-MHC CaMKKβ^kd^ TG mice by immunoprecipitate kinase assays.

It is known that mechanical overload evokes a Ca^2+^-mediated signaling cascade in the heart. Moreover, CaMKK is activated by the elevation of intracellular Ca^2+^ and it may transduce important signals during cardiac hypertrophy. Thus, we first examined the expression of CaMKKβ in the course of cardiac hypertrophy induced by TAC. We found that the mRNA and protein levels of CaMKKβ were significantly increased 1 week after TAC and continued to increase gradually thereafter (Fig. 1A).

### 2. Generation of α-MHC CaMKKβ^kd^ TG mice

To study the impact of CaMKKβ expression on pressure-loaded heart failure, we generated α-MHC CaMKKβ^kd^ TG mice. A catalytically inactive mutant of CaMKKβ was made by mutating aspartic acid 329 to alanine (D329A) [7], [9], [15]. The cardiomyocyte-specific α-MHC promoter has a transient burst of activity in embryonic hearts between embryonic day 9.5 and 10.5, and its activity is reactivated during early postnatal life and remains persistently high into adulthood [16]. We obtained three independent transgenic lines. The results from two lines are shown in Fig. 1. Western blotting using anti-FLAG and anti-CaMKKβ antibodies confirmed cardiac overexpression and the levels of CaMKKβ^kd^ transgenes in α-MHC CaMKKβ^kd^ TG mice (Fig. 1B and C). We also determined the activities of CaMKKβ in the hearts of these mice using immunoprecipitate kinase assays (Fig. 1D). CaMKKβ activity was significantly suppressed in the two lines of α-MHC CaMKKβ^kd^ TG mice. These transgenic mice appeared to have normal cardiac development and life span (survival of over 12 months). There was no change in body weight, systolic blood pressure, or heart rate in intermediate and high expression lines of α-MHC CAMKKβ^kd^ TG mice.

### 3. α-MHC CaMKKβ^kd^ TG mice had higher mortality compared with WT mice

**Figure 2:**
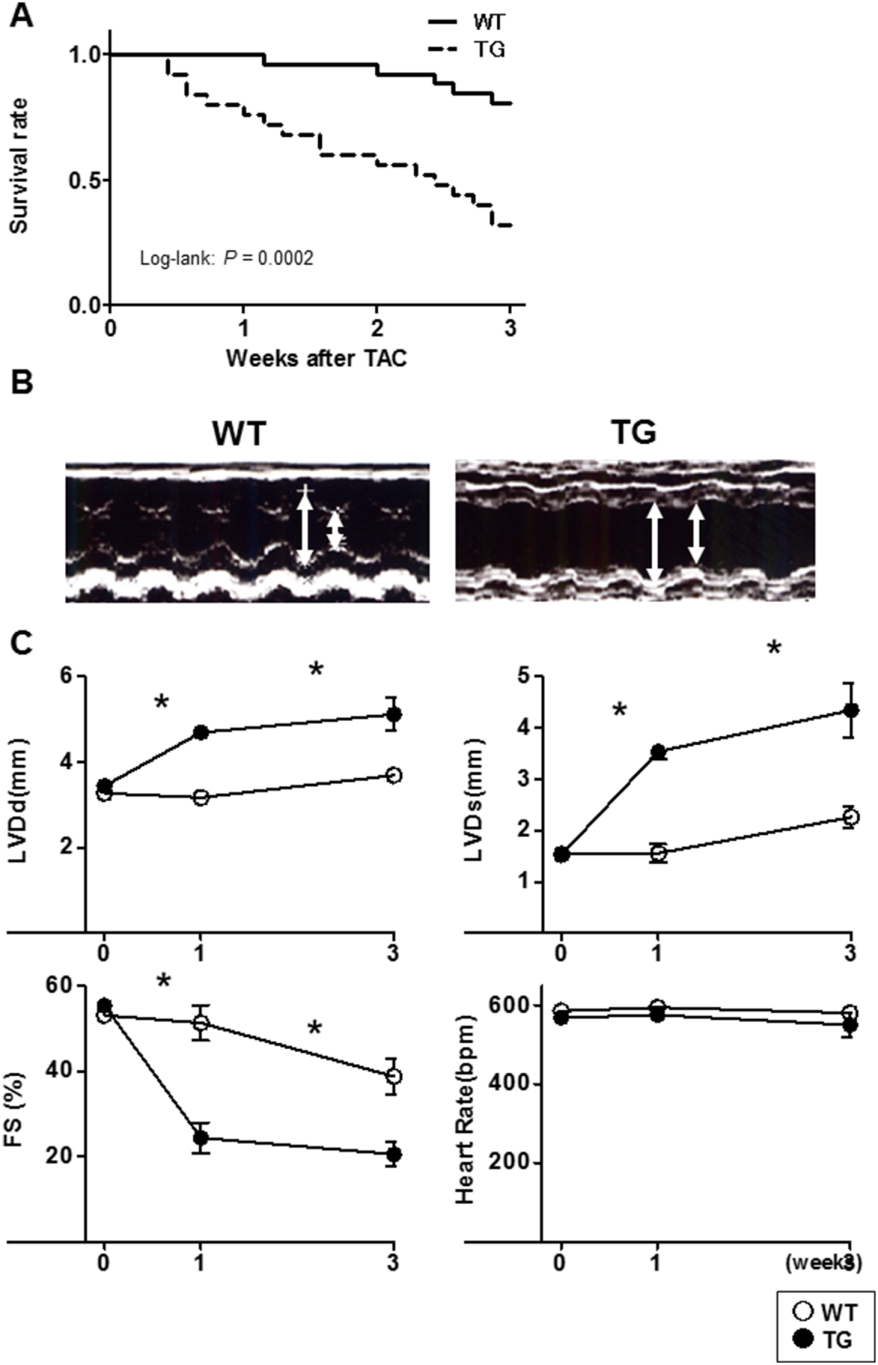
Kaplan-Meier plot of cardiac failure-free fraction and cardiac function of WT and α-MHC CaMKKβ^kd^ TG mice after TAC. A. α-MHC CaMKKβ^kd^ TG mice had a lower cardiac failure-free fraction compared with WT mice. Log-rank test indicates the significant difference between WT and α-MHC CaMKKβ^kd^ TG mice after TAC (p = 0.0002) (WT; n = 26, TG; n = 25). B. Representative echocardiograph pictures of WT and α-MHC CaMKKβ^kd^ TG hearts 3 weeks after TAC. Arrows indicate left ventricular cavities of diastolic and systolic phase. C. Serial changes in echocardiographic parameters of WT and α-MHC CaMKKβ^kd^ TG mice after TAC. *p<0.05 vs WT (n = 3–5 for each group).

**Figure 3:**
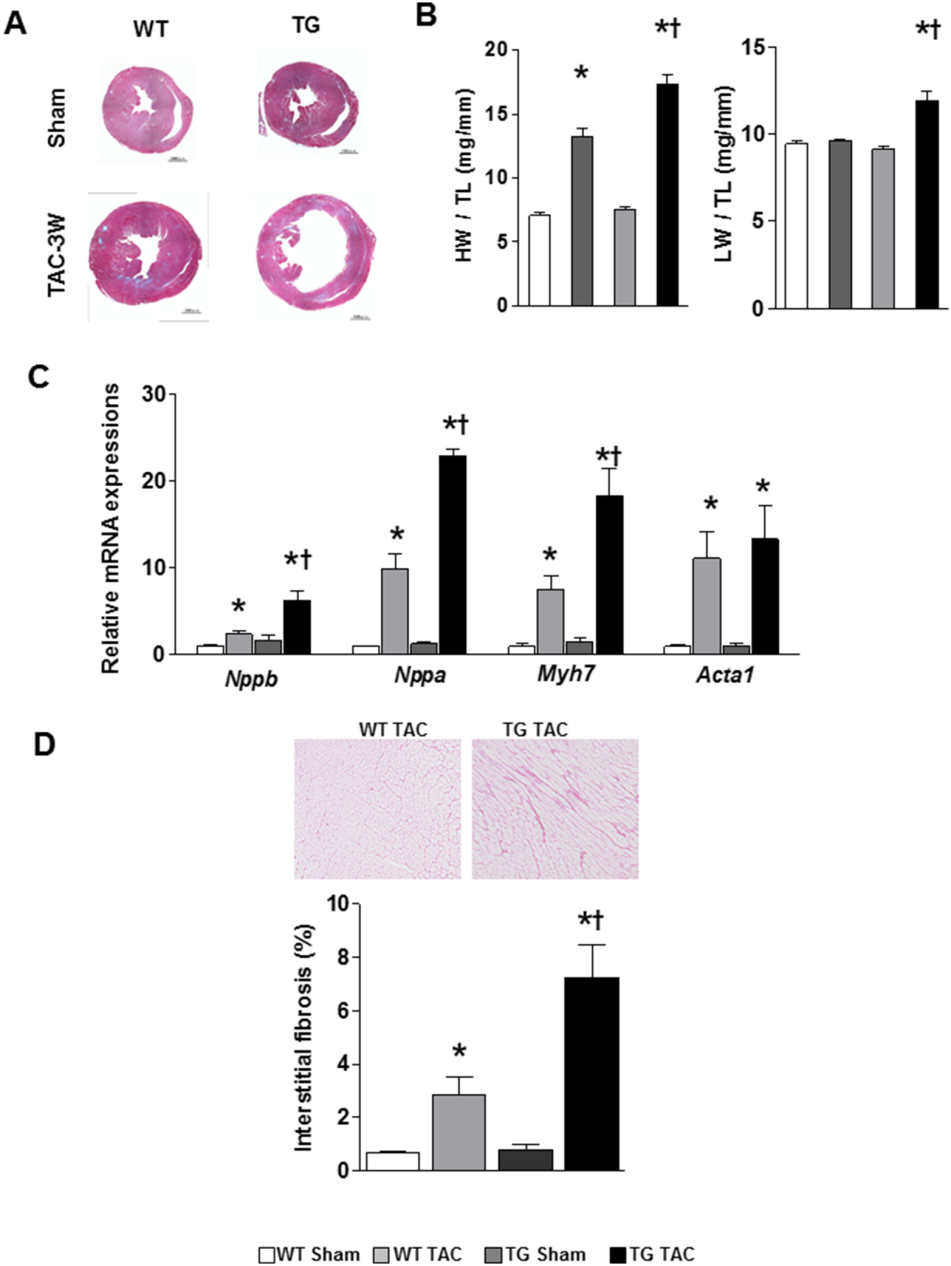
Left ventricular dilatation, hypertrophy, and fibrosis is prominent in α-MHC CaMKKβ^kd^ TG mice compared with WT mice after TAC. A. Low-power magnifications of Masson Trichrome stained images of the left ventricle of WT and α-MHC CaMKKβ^kd^ TG mice with or without TAC. B. Heart weight (HW) and lung weight (LW) of WT and α-MHC CaMKKβ^kd^ TG mice with or without TAC. Each value was normalized to tibial length (TL). Values are the means ± S.E. of 5 mice (*p<0.05 vs sham control, †p<0.05 vs WT after TAC). C. Relative expression levels of hypertrophy-associated genes in WT and α-MHC CaMKKβ^kd^ TG mice with or without TAC. Values are the means ± S.E. of four mice (*p<0.05 vs sham control, †p<0.05 vs WT after TAC). D. Picrosirius staining of the left ventricle in WT and α-MHC CaMKKβ^kd^ TG mice with or without TAC and measurement the area of fibrosis. Values are the means ± S.E. of three to five mice (*p<0.05 vs sham control, †p<0.05 vs WT after TAC).

Three-month-old α-MHC CaMKKβ^kd^ TG lines and WT control mice underwent TAC and were followed over the next 8 weeks. According to the humane endpoint rules, mice subjected to TAC had to be sacrificed when the criteria of endpoints were reached. Using such criteria, Kaplan-Meier analysis demonstrated that α-MHC CaMKKβ^kd^ TG mice were significantly more likely to develop this degree after TAC than WT control mice (Fig. 2A). To determine the functional aspects of α-MHC CaMKKβ^kd^ TG hearts, we used an echocardiograph to analyze cardiac structure and function after TAC. Fig. 2B shows representative results of echocardiography of the heart of WT mice and the high expression line of α-MHC CaMKKβ^kd^ TG mice at 3 weeks after TAC. The other line of TG mice showed the same results. Fig. 2C demonstrates that α-MHC CaMKKβ^kd^ TG mice had a significant increase in LV end diastolic diameter (LVDd) and LV systolic diameter (LVDs), accompanied by a reduction in contraction measured as fractional shortening (FS). Further experiments were carried on the high expression line. Fig. 3A contains low-power magnifications of Masson trichrome staining of the heart, which confirmed that marked dilatation occurred in the hearts of α-MHC CaMKKβ^kd^ TG mice subjected to TAC compared with both WT TAC hearts and α-MHC CaMKKβ^kd^ TG hearts without TAC. Hearts from WT mice subjected to TAC were enlarged and their weight increased twofold compared with those from WT mice without TAC (Fig. 3B), which was consistent with cardiac hypertrophy. In contrast, hearts from α-MHC CaMKKβ^kd^ TG mice almost tripled in weight compared with those from α-MHC CaMKKβ^kd^ TG mice without TAC (Fig. 3B) and the lungs in α-MHC CaMKKβ^kd^ TG mice also increased in weight, probably resulting from extreme congestion, compared with those in WT TAC mice. mRNA expression levels of both atrial and B-type natriuretic factor (*Nppa* and *Nppb*), heavy chain of β myosin (*Myh7*), and α1 actin (*Acta1*), which are markers of cardiac hypertrophy and failure, were increased significantly in WT mice after TAC compared with mice without TAC and in α-MHC CaMKKβ^kd^ TG mice after TAC compared with WT mice after TAC (Fig. 3C). Picrosirius staining indicated that interstitial fibrosis increased significantly in WT mice after TAC compared with mice without TAC, and the levels increased significantly in α-MHC CaMKKβ^kd^ TG mice after TAC compared with WT mice after TAC (Fig. 3D).

### 4. Altered CaMKKβ-mediated signaling in α-MHC CaMKKβ^kd^ TG hearts after TAC

**Figure 4:**
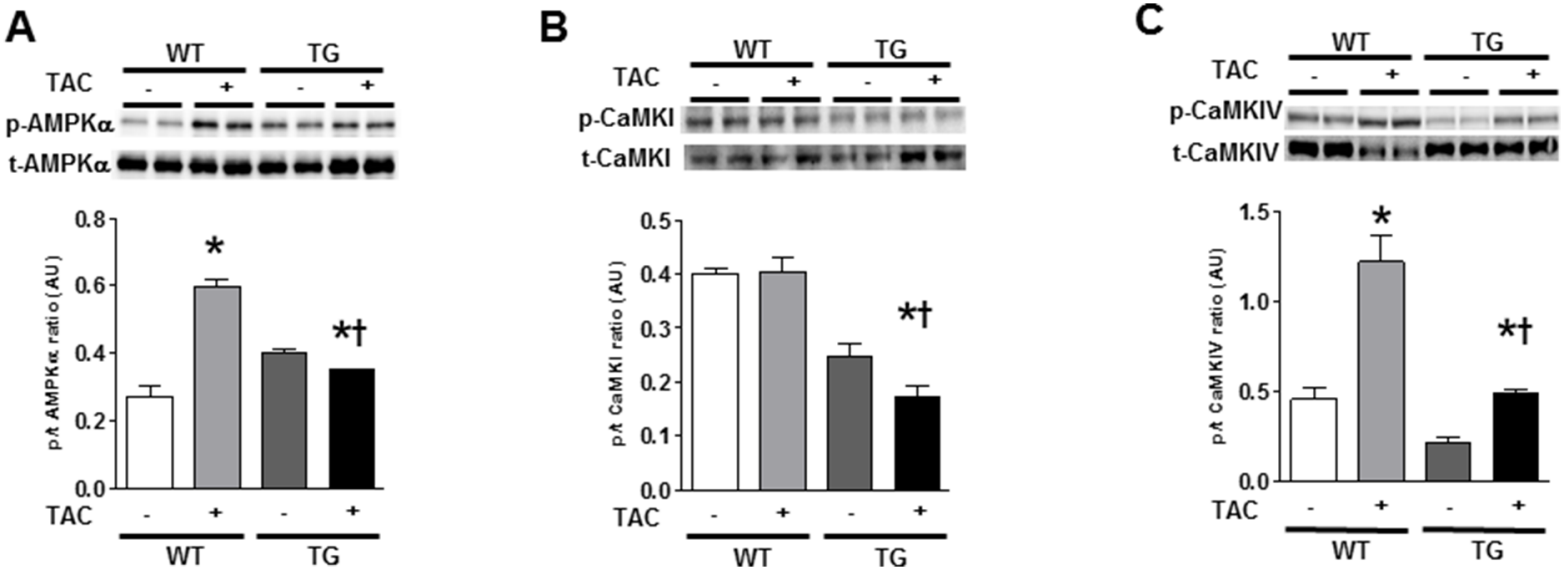
Phosphorylation of downstream targets of CaMKKβ in WT and α-MHC CaMKKβ^kd^ TG mice with or without TAC. Representative pictures of immunoblotting for phosphorylated form and total amount of adenosine monophosphate (AMP)-activated protein kinase (AMPK), calcium/calmodulin-dependent protein kinase (CaMK) I, and CaMKIV (A–C). Results of densitometric analysis are indicated. Values are the means ± S.E. of five mice (*p<0.05 vs sham control, †p<0.05 vs WT after TAC).

Next, we measured the phosphorylation levels of the AMPK, CaMKI, and CaMKIV, which are well-established CAMKKβ downstream targets, in the hearts of WT and α-MHC CaMKKβ^kd^ TG mice after sham surgery or TAC. Figs. 4A to C show the results of Western blotting and densitometry analysis of the blots. The phosphorylation levels increased significantly in WT hearts after TAC except for CaMKI; however, the phosphorylation levels of AMPK, CaMKI, and CaMKIV were significantly reduced in α-MHC CaMKKβ^kd^ TG hearts compared with the WT mice after TAC.

### 5. Abnormal morphology of mitochondria and mitochondrial gene expression in α-MHC CaMKKβ^kd^ TG hearts after TAC

**Figure 5:**
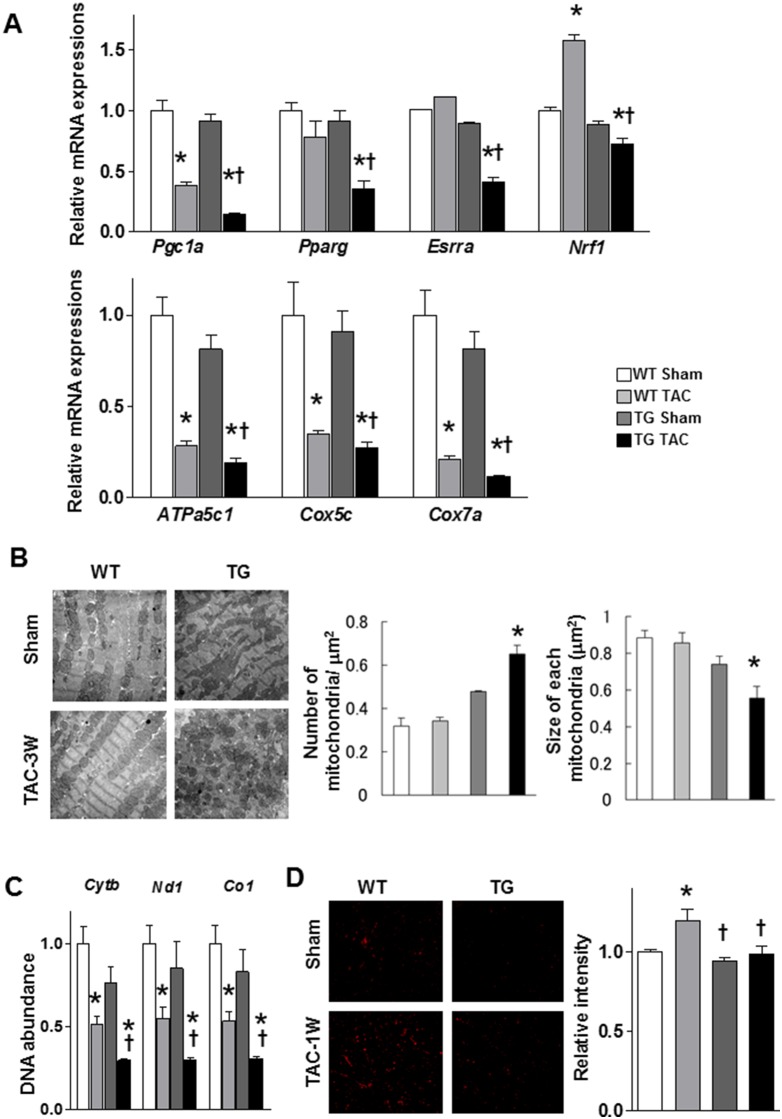
Mitochondrial ultrastructure, DNA abundance, and metabolism-related gene expression levels in WT and α-MHC CaMKKβ^kd^ TG mice with or without TAC. A. Relative mRNA expression levels related to mitochondrial function and biogenesis. Values are the means ± S.E. of six mice (*p<0.05 vs sham control, †p<0.05 vs WT after TAC). B. Abnormal mitochondrial ultrastructure in α-MHC CaMKKβ^kd^ TG hearts after TAC. C. The amount of mitochondrial DNA quantified by quantitative PCR. Cytochrome b (*Cytb*), NADH dehydrogenase subunit 1 (*Nd1*) and cytochrome c oxidase 1 (*Co1*) expression levels relative to nuclear DNA in the ventricles of WT and α-MHC CaMKKβ^kd^ TG mice with or without TAC are shown. Values are the means ± S.E. of three to four mice (*p<0.05 vs sham control, †p<0.05 vs WT after TAC). D. Superoxide levels detected using a superoxide-sensitive dye, dihydroethidine (DHE). Representative pictures are shown on the left. Relative intensities are shown on the right. Values are the means ± S.E. of three to six mice (*p<0.05 vs sham control, †p<0.05 vs WT after TAC).

The expression of PGC-1α has been reported to be modulated in several physiological contexts by increased Ca^2+^ signaling via molecules such as CaMK and CREB [17] and by CaMKKβ [8]. Therefore, we measured PGC-1α expression levels in the left ventricles of WT and α-MHC CaMKKβ^kd^ TG mice with or without TAC. As shown in Fig. 5A, *PGC1a* expression levels were the same in sham-operated WT and α-MHC CaMKKβ^kd^ TG mice; however, its expression was significantly reduced in α-MHC CaMKKβ^kd^ TG hearts compared with WT mice after TAC. *Pparg*, *Esrrsa*, *and Nrf1* gene expression levels were significantly reduced in α-MHC CaMKKβ^kd^ TG hearts compared with WT mice after TAC. We further measured *ATPa5c1*, *Cox5c*, *and Cox7a* gene expression levels. These levels also showed the same pattern as those of *PGC1a*. We then observed changes in mitochondrial morphology using electron microscopy. As shown in Fig. 5B, the mitochondria of α-MHC CaMKKβ^kd^ TG mice were the same as those of WT mice before TAC; however, the size of mitochondria in α-MHC CaMKKβ^kd^ TG mice became smaller than those of WT mice after 3 weeks’ TAC. We then measured the amount of mitochondrial DNA by PCR analysis of *Cytb*, *Nd1*, and *Co1* genes. Fig. 5C indicates that the levels of these genes were significantly decreased after TAC in both WT and α-MHC CaMKKβ^kd^ TG mice compared with those mice before TAC, and the levels were further decreased after TAC in α-MHC CaMKKβ^kd^ TG mice compared with WT mice after TAC. Then, we measured superoxide levels using the superoxide-sensitive dye DHE. The density of DHE increased in WT hearts after TAC; however, such an increase was not observed in α-MHC CaMKKβ^kd^ TG hearts after TAC compared with that before TAC. All of these data show an abnormality in mitochondria-related gene expression patterns and their function in α-MHC CaMKKβ^kd^ TG hearts after TAC.

### 6. Impaired energetics and ATP depletion in α-MHC CaMKKβ^kd^ TG hearts after TAC

**Figure 6:**
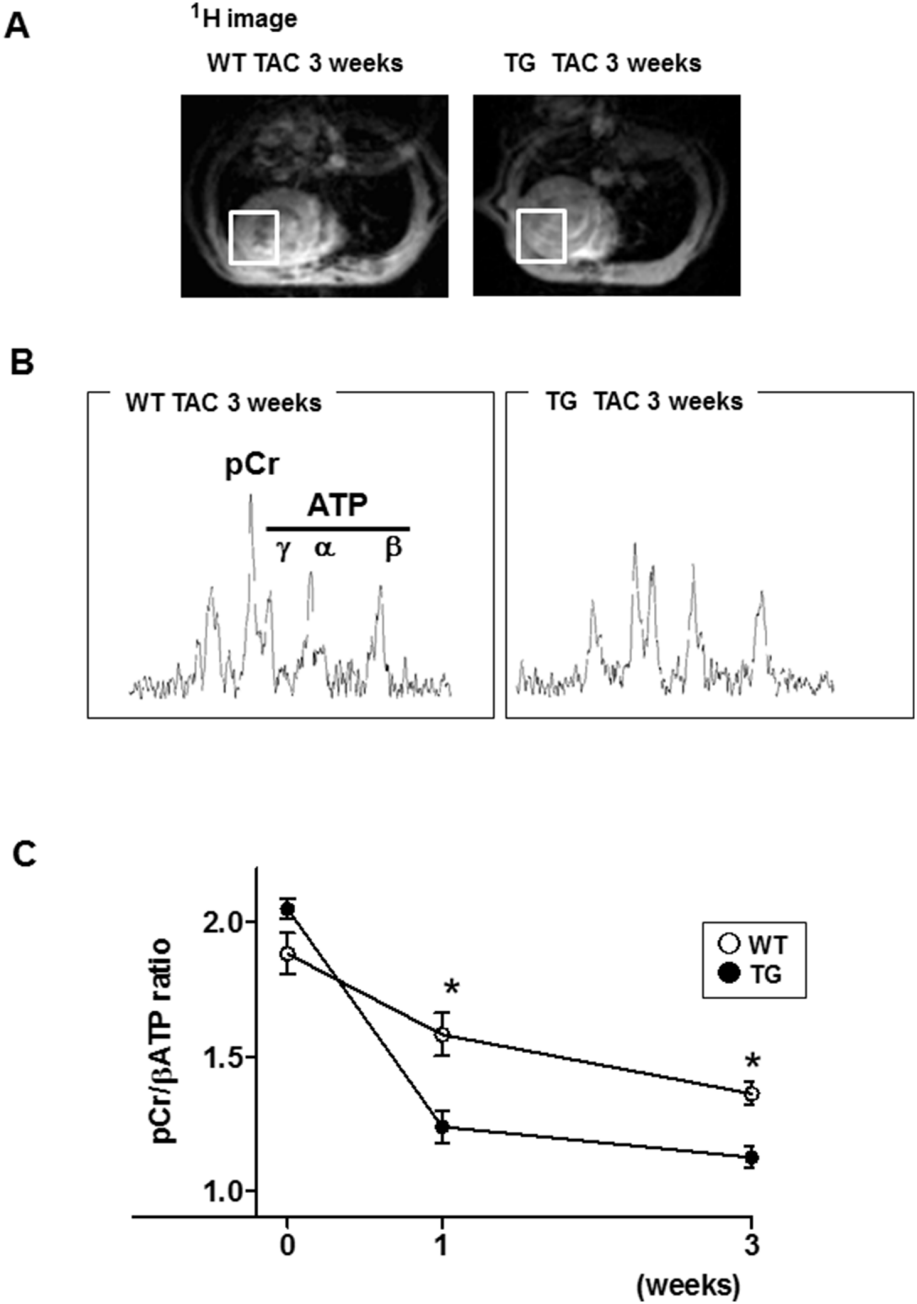
Myocardial energy reserve measured by in situ ^31^P magnetic resonance (MR) spectroscopy. A. A ^1^H MR image to define the region of interest to measure a ^31^P MR spectrum of the left ventricular anterior wall. B. In vivo cardiac ^31^P MR spectra from WT and α-MHC CaMKKβ^kd^ TG mice 3 weeks after TAC. C. The creatine phosphate/ATP ratio of the left ventricle in WT and α-MHC CaMKKβ^kd^ TG after TAC. Values are mean±standard error of the mean. *p<0.05 vs WT at same time point, n = 3–8 for each group.

We further tried to measure the changes in energy levels in the hearts of WT and α-MHC CaMKKβ^kd^ TG mice in response to pressure load. ^31^P MR spectrometry offers a unique means to noninvasively quantify the major cardiac high-energy phosphates PCr and ATP, which are critical for normal myocardial contractile function and viability. We used ^31^P MR spectrometry to quantify the *in vivo* PCr-to-ATP ratio (PCr/ATP) of murine hearts. Fig. 6A shows a ^1^H MR image to define the region of interest to measure the ^31^P MR spectrum of the LV anterior wall. Representative cardiac ^31^P MR spectra from mice are shown in Fig. 6B. As shown in Fig. 6C, the PCr/ATP ratio of α-MHC CaMKKβ^kd^ TG mice was the same as that of WT before TAC. The mean in vivo myocardial PCr/ATP ratios were 2.05±0.04 and 1.88±0.08, respectively, which were consistent with previous reports [18]. TAC significantly reduced mean PCr/ATP ratio to 1.58±0.08 in WT mice; however, the level was significantly lower in α-MHC CaMKKβ^kd^ TG mice (1.24±0.06) as early as 1 week after TAC compared with that of WT mice. The difference between WT and α-MHC CaMKKβ^kd^ TG mice remained unchanged until 3 weeks after TAC.

## Discussion

We have reported previously that CaMKKβ can activate AMPK in cardiomyocytes and is involved in GLUT4 translocation, based on the finding that the CaMKK inhibitor STO-609, as well as overexpression of the dominant-negative form of CaMKKβ, inhibited its H_2_O_2_-mediated translocation [7]. It was also suggested that H_2_O_2_-mediated increase in the intracellular Ca^2+^ concentration is most likely to play a more important role than increase in AMP:ATP ratio in AMPK activation [19]. In this experiment, we tried to find out the mechanism of cardiac energy production against sustained pressure overload by the use of α-MHC CaMKKβ^kd^ TG mice, because the Ca^2+^-mediated signaling cascade is important in this situation. The major findings of this study are as follows: (1) CaMKKβ expression was increased in the left ventricle in response to pressure-overload stress by TAC in WT mice; (2) TAC in α-MHC CaMKKβ^kd^ TG mice resulted in a significant inhibition of CaMKKβ downstream signaling molecules, including AMPK, compared with those in WT mice and led to accelerated cardiac dysfunction, which was accompanied by signs of significant clinical heart failure and death; and (3) the expression levels of PGC-1α, which is a downstream target of both of CaMKKβ and CaMKs, were also significantly reduced in α-MHC CaMKKβ^kd^ TG mice compared with WT mice after TAC. In accordance with these findings mitochondrial morphogenesis was damaged and PCr/β-ATP ratios assessed by MR spectroscopy were also suppressed in α-MHC CaMKKβ^kd^ TG mice compared with WT mice after TAC. To the best of our knowledge, these findings provide the first evidence that CaMKKβ exerts an effect on cardiac adaptive energy pooling against pressure-overload-induced ventricular dysfunction.

Heart failure is a multifactorial, progressive, and disabling syndrome leading to a deterioration of the heart characterized by a symptoms resulting from ventricular dysfunction. Contractile dysfunction is often linked to chronic energy deficit. The increased wall stress of the ventricle enhances local oxygen consumption and worsens the energy deficiency and function. As a consequence, the heart enters a vicious cycle. In this context, alternative therapies that could improve the energetic state and disrupt the vicious cycle of the failing heart are of particular interest.

We hypothesized that there may be a signaling mechanism to compensate cardiac energy production against sustained pressure load. Previous studies explain that the Ca^2+^-mediated signaling cascade induced by mechanical overload or Gq-mediated signaling initiates the changes that lead to cardiac hypertrophy [20]. CaMKKβ may be one such molecule that links Ca^2+^ signaling and cardiac metabolism, because it is involved in regulating many important physiological processes, including energy balance, adiposity, glucose homeostasis, and hematopoiesis [21].

Our results indicated that phosphorylation of AMPK, CaMKI, and CaMKIV was reduced in α-MHC CaMKKβ^kd^ TG mice compared with WT mice after TAC. AMPK plays an important role in regulating energy balance, protein synthesis, and cell growth. AMPK activation enhances fatty acid and glucose metabolism to augment ATP production and attenuates protein synthesis to preserve ATP. We indicated previously that AMPK is important for oxidative stress-mediated GLUT4 translocation in cardiomyocytes [7]. This pathway may be enhanced under mechanical overload with the elevation of oxidative stress [22]. Moreover, because the energy substrate switches from fatty acids to glucose under cardiac hypertrophy/heart failure [23], [24], [25], the reduction in AMPK phosphorylation may have negatively affected energy supply in α-MHC CaMKKβ^kd^ TG mice compared with WT mice after TAC. This may be one of the reasons why PCr/β-ATP ratios assessed by MR spectroscopy were suppressed in α-MHC CaMKKβ^kd^ TG mice compared with WT mice after TAC.

Previously, Zhang et al. reported that TAC resulted in more hypertrophy and fibrosis in AMPKα2-deficient hearts than in WT hearts, with a greater increase in LV diameter at end systole and a greater decrease in LV ejection fraction [26]. Although there is a difference in LV phenotype between the study by Zhang et al. and the present study, it may be simply because other downstream target of CaMKKβ such as CaMKI and CaMKIV affected LV structure and function in this experiment.

The expression of PGC-1α has been reported to be modulated in several physiological contexts, for example, in skeletal muscle in response to exercise partly by increased Ca^2+^ signaling via molecules such as CaMK and CREB [17]. A recent study indicated that quiescent and noradrenaline-exposed CaMKKβ-null hepatocytes express less mRNA encoding PGC-1α compared with WT hepatocytes [27]. In line with these findings, CaMKKβ was shown to be important for mitochondrial biogenesis and exercise tolerance through the activation of its downstream target PGC-1α by the use of muscle-specific adiponectin-deficient mice [8]. Therefore, we measured PGC-1α levels and found that its level was significantly reduced in α-MHC CaMKKβ^kd^ TG mice compared with WT mice after TAC. Not only PGC-1α, but other mitochondrial biogenesis genes were dysregulated, and morphology and function also deteriorated in α-MHC CaMKKβ^kd^ TG mice compared with WT mice after TAC. It is known that PGC-1α is abundantly expressed in the heart and a number of gain-of-function and loss-of-function assays have shown that PGC-1α activates most genes of mitochondrial biology and stimulates both fatty acid oxidation and oxidative respiration in cardiac tissues [28], [29], [30], [31], [32]. The decrease in PGC-1α expression and mitochondrial morphological changes would be another reason for the reduction in energy pooling in the left ventricle of α-MHC CaMKKβ^kd^ TG mice. These data indicate the vital role that PGC-1α plays in maintaining normal cardiac energetics and contractile function, especially in response to physiological stimuli.

Our data clearly indicate that CaMKKβ exerts beneficial effects against pressure-overload-induced heart failure. It is important to note, however, that massive induction of PGC-1α in the heart increased mitochondrial content to such an extent that myofibrils were displaced and resulted in cardiomyopathy [28], [31]. Hence, it may be necessary to increase CaMKKβ activity in an appropriate and moderate fashion for the treatment of heart failure.

## Supporting Information

Table S1 **Primer sequences used for Quantification of mRNA and DNA levels.**
(DOC)

Checklist S1 **ARRIVE Checklist.**
(PDF)
