# Supplementary material for: Cardiac-Specific Inhibition of Kinase Activity in Calcium/Calmodulin-Dependent Protein Kinase Kinase-β Leads to Accelerated Left Ventricular Remodeling and Heart Failure after Transverse Aortic Constriction in Mice
Source: PLoS One. 2014 Sep 25;9(9):e108201. doi: 10.1371/journal.pone.0108201 (PMC4177887; doi:10.1371/journal.pone.0108201)
Supplement: Table S1 — Primer sequences used for Quantification of mRNA and DNA levels. (DOC) [file pone.0108201.s001.doc]

**Supplemental information**

| **Supplementary Table.** Primer sequences used for Quantification of mRNA and DNA levels | | |
| --- | --- | --- |
|  | Forward primer | Reverse primer |
| *Nppb* | GCCAGTCTCCAGAGCAATTCA | TGTTCTTTTGTGAGGCCTTGG |
| *Nppa* | ATGGGCTCCTTCTCCATCAC | ATCTTCGGTACCGGAAGCTG |
| *Myh7* | GCCAACACCAACCTGTCCAAGTTC | TGCAAAGGCTCCAGGTCTGAGGGC |
| *Acta1* | TATTCCTTCGTGACCACAGCTGAACGT | CGCGAACGCAGACGCGAGTGCGC |
| *PGC1a* | TTCTGGGTGGATTGAAGTGGTG | TGTCAGTGCATCAAATGAGGGC |
| *Pparg* | ATGGAACAGCCACAGGAGGA | ATCACAGCCCATCTGCAGCT |
| *Esrra* | CAAGAGCATCCCAGGCTT | GCACTTCCATCCACACACTC |
| *Nrf1* | GAACTGCCAACCACAGTCAC | TTTGTTCCACCTCTCCATCA |
| *Atp5c1* | TCAAGTCTGTTATCTCCTAC | GAGGTTGGCCAGATTGTAC |
| *Cox5a* | TGGAGGTGGTGTCCCTACTG | CTCTTGTTGCTGATGGATGG |
| *Cox7a1* | ATGAGGGCCCTACGGGTCTC | CATTGTCGGCCTGGAAGAG |
| *H19* | CCGCTCAACCACCTAATTGT | TGGGTGGGAGAGAATGAGAC |
| *Cytb* | ATTCCTTCATGTCGGACGAG | ACTGAGAAGCCCCCTCAAAT |
| *Nd1* | CCTTCGACCTGACAGAAGGA | GATGCTCGGATCCATAGGAA |
| *Co1* | GCCTTTCAGGAATACCACGA | AGGTTGGTTCCTCGAATGTG |

Nppb; natriuretic peptide B, Nppa; natriuretic peptide A, Myh7; myosin, heavy chain 7, Acta1; actin, alpha 1, skeletal muscle, PGC1a; peroxisome proliferator activated receptor gamma coactivator 1 alpha, Pparg; peroxisome proliferator activated receptor gamma, Esrra; estrogen related receptor, alpha, Nrf1; nuclear respiratory factor 1, Atp5c1; ATP synthase, H+ transporting, mitochondrial F1 complex, gamma polypeptide 1, Cox5a; cytochrome c oxidase subunit Va, Cox7a1; cytochrome c oxidase subunit VIIa 1, H19; imprinted maternally expressed transcript (non-protein coding), Cytb; cytochrome b, Nd1; NADH dehydrogenase subunit 1, Co1; cytochrome c oxidase 1
